# Supplementary material for: Systematic Review of Intervention Studies Aiming at Reducing Inequality in Dental Caries among Children
Source: Int J Environ Res Public Health. 2021 Feb 1;18(3):1300. doi: 10.3390/ijerph18031300 (PMC7908536; doi:10.3390/ijerph18031300)
Supplement: Supplementary file 1 [file ijerph-18-01300-s001.pdf]

Supplementary File1 MESH words, search string and search terms

Search: **((((((dental caries) OR (decayed teeth)) OR (cavities)) AND (child)) AND (socioeconomic)) OR (inequalities)) OR (disparities)** Filters: **Clinical Trial, Randomized Controlled Trial, Humans, Chinese, English, Child: birth-18 years**

(("dental caries"[MeSH Terms] OR ("dental"[All Fields] AND "caries"[All Fields]) OR "dental caries"[All Fields] OR ("dental caries"[MeSH Terms] OR ("dental"[All Fields] AND "caries"[All Fields]) OR "dental caries"[All Fields] OR ("decayed"[All Fields] AND "teeth"[All Fields]) OR "decayed teeth"[All Fields]) OR ("cavity s"[All Fields] OR "dental caries"[MeSH Terms] OR ("dental"[All Fields] AND "caries"[All Fields]) OR "dental caries"[All Fields] OR "cavities"[All Fields] OR "cavity"[All Fields])) **AND** ("child"[MeSH Terms] OR "child"[All Fields] OR "children"[All Fields] OR "child s"[All Fields] OR "children s"[All Fields] OR "childrens"[All Fields] OR "childs"[All Fields]) **AND** ("socioeconomic factors"[MeSH Terms] OR ("socioeconomic"[All Fields] AND "factors"[All Fields]) OR "socioeconomic factors"[All Fields] OR "socioeconomics"[All Fields] OR "socioeconomic"[All Fields] OR "socioeconomical"[All Fields] OR "socioeconomically"[All Fields])) OR ("inequalities"[All Fields] OR "inequality"[All Fields] OR "inequities"[All Fields] OR "inequity"[All Fields]) OR ("disparate"[All Fields] OR "disparately"[All Fields] OR "disparities"[All Fields] OR "disparity"[All Fields])

dental caries: "dental caries"[MeSH Terms] OR ("dental"[All Fields] AND "caries"[All Fields]) OR "dental caries"[All Fields]

decayed teeth: "dental caries"[MeSH Terms] OR ("dental"[All Fields] AND "caries"[All Fields]) OR "dental caries"[All Fields] OR ("decayed"[All Fields] AND "teeth"[All Fields]) OR "decayed teeth"[All Fields]

cavities: "cavity's"[All Fields] OR "dental caries"[MeSH Terms] OR ("dental"[All Fields] AND "caries"[All Fields]) OR "dental caries"[All Fields] OR "cavities"[All Fields] OR "cavity"[All Fields]

child: "child"[MeSH Terms] OR "child"[All Fields] OR "children"[All Fields] OR "child's"[All Fields] OR "children's"[All Fields] OR "childrens"[All Fields] OR "childs"[All Fields]

socioeconomic: "socioeconomic factors"[MeSH Terms] OR ("socioeconomic"[All Fields] AND "factors"[All Fields]) OR "socioeconomic factors"[All Fields] OR "socioeconomics"[All Fields] OR "socioeconomic"[All Fields] OR "socioeconomical"[All Fields] OR "socioeconomically"[All Fields]

inequalities: "inequalities"[All Fields] OR "inequality"[All Fields] OR "inequities"[All Fields] OR "inequity"[All Fields]

disparities: "disparate"[All Fields] OR "disparately"[All Fields] OR "disparities"[All Fields] OR "disparity"[All Fields]

Supplementary File2 Summary of intervention studies on reducing inequality in dental caries

Author/year/ Country	Sampling (Sample size/ age group)	Follow-up duration	Intervention group	Comparison group	Outcomes	Results and Conclusions
Shin (2020) South Korea	4353 at baseline and 2915 at follow-up. (2240 children aged 6-11years old and 2113 children aged 12-18 years old at baseline, and 1449 children aged 6-11years old and 1466 children aged 12-18 years old at follow-up)	6 years	The children and adolescents were separated to receive **dental sealants** during 6 years follow-up.	No dental sealants in the comparison group.	Untreated dental caries and sealant experience. Trained and calibrated dentists conducted the oral health examination. However, no dental index used was mentioned.	This study found that the National health coverage expansion of dental care had a **positive effect** on overall status in dental health among children and adolescents.<br><br>The adjusted prevalence ratios of untreated dental caries and sealants in the poorest in the aged 6–11 group were significantly higher and lower, respectively, compared to the most affluent quartile group for the baseline. However, all significant differences disappeared for the follow-up after the sealant coverage. The gap between the lowest and the highest was similar for the aged 12–18 group but it widened in the untreated dental caries even after the sealant coverage.
Winter (2018) Germany [14]	1079 children aged 7 years old at baseline, and 832 children aged 9 years old at follow-up.	2 years	1.Intensive prevention: based on tooth-brushing with fluoride content 12,500 ppm) under supervision of a TBF at intervals of 3 weeks during school sessions<br><br>2. Basic prevention: received	No organized prevention in kindergarten.	Caries increment (ICDAS); DMFT/DMFS index; Sulcus bleeding index (SBI) for periodontal tissues.	Early prevention, focusing on training of toothbrushing in kindergarten and at school, has a positive effect on dental health and is able to **reduce** class-specific differences in caries distribution.<br><br>A significant difference was found in the mean DMFT depending on socioeconomic status (no prevention in

			instruction on tooth-brushing three to four times a year and a free supply of toothpaste with a fluoride content of 1400 ppm for use at home.			kindergarten, fluoride gel at school in children with low SES: DMFT = 0.47 vs. DMFT = 0.18 in children with high SES; p = 0.023).
Qadri (2018) Germany [15]	Baseline: 854 children at grade 5 (401 in intervention and 453 in controlled group); Follow-up: 740 children at grade 6 (336 in intervention and 404 in controlled group)	19 months	General and **oral health education** was provided to the teachers in the intervention schools, which they then conveyed to their students. This covered oral health, healthy nutrition, health literacy, dealing with pain, healthy recreation (activities and leisure time), vaccination and smoking.	No additional educational program or training on general health promotion in the control group.	Dental caries was expressed using DMFT index. SES inequality was compared using education, vocational training, net household income and employment status.	School-based oral health education **increases** caries inequalities. The program was effective in improving dental health among students with higher SES.<br><br>A significant incident rate ratio between caries increment was found, with a 35% higher risk in the control group. However, parents' SES modified the effect of the program on their children, as high SES in the intervention group was associated with 94% reduction in the incidence risk ratio ($p < 0.001$).
Matsuyama (2016) Japan [10]	**47 prefectures** were selected in the study. Baseline: 7 years old children at baseline and 12 years old children at follow-up.	5 years	The **school-based fluoride mouth-rinse** program (S-FMR) was the proportion of children in the prefecture who received S-FMR, obtained from the National Survey on School-based Fluoride Mouth-rinse Programs in Japan.	No intervention	DMFT index was used to reflect the population benefit of S-FMR. Dental caries status at ages 3 and 12 years were obtained from dental health	Utilization of S-FMR **reduced** area-based deprivation inequalities in dental caries by means of proportionate universalism.<br><br>High S-FMR utilization was significantly associated with low DMFT at age 12 (coefficient −0.011; 95% CI: −0.018, −0.005). S-FMR utilization explained 25.2% of the DMFT variance between prefectures after considering other variables. S-FMR, administered to

			Other variables: fluoride toothpaste, dentist density, average sugar consumption per capita in each prefecture and mean annual income		checkups.	children of all socioeconomic status, was associated with lower DMFT.
Tubert-Jeannin (2012) France [20]	All the 5 years old children attending 21 selected schools (n=620) at baseline and 478 children at follow-up	5 years	**The oral health promotion program** included various educational activities conducted with the carers (parents, teachers, school nurses) of the children. Guidelines promoting oral health (oral hygiene, nutrition, dental care) were developed and circulated to carers.	No intervention	Carious lesions were recorded in dmft and dmfs index using ICDAS criteria. SES was classified as deprived, semi-deprived and non-deprived.	The OHP program **did not reduce school-deprivation** inequalities in oral health, even if dental status improved in four schools (**no effect**).<br><br>Caries experience varied significantly with deprivation status, oral hygiene and household SES indicators. The only difference observed between 2003 and 2009 was an increase in the 'f' component ($p < 0.001$). Dental status had slightly deteriorated in areas characterized in 2003 by low caries levels (p=0.07). In deprived areas, mean dmft increased in schools without the OHP program ($p = 0.04$).
Plutzer (2010) Australia [16]	441 children aged 6 at baseline and 426 children aged 20 months at follow-up.	14 months	The intervention was applied during pregnancy and child aged 6 and 12 months old. The intervention consisted of three rounds of printed **oral health promotion** material. Mothers also received a small incentive,	No intervention	Children in both groups were examined for the presence of S-ECC at the age of 20 months. S-ECC was defined as one or	The intervention produced a greater **reduction** in the frequency of ECC in children from one-parent families than in those from two-parent families. Children in one-parent families had a four times higher risk than children from two-parent families.<br><br>The intervention reduced the frequency of ECC from

			consisting of mouth rinse, toothbrush or finger toothbrush for the child, to boost the information. The intervention was enhanced through telephone counselling, when the children were between 6 and 12 months old, in half of the mothers (randomized again) in the intervention group.		more upper anterior incisor carious. No dental criteria mentioned in this study.	8.1% to 1.1% in two-parent families (relative risk: 0.14) and from 16.3% to 4.5% (relative risk: 0.28) in one-parent families. Despite a greater reduction in the absolute risk of ECC in children from one-parent families, the intervention reduced their ECC experience only 3.5-fold compared with sevenfold in children from two-parent families.
Meurman (2009) Finland [19]	1128 children aged 18 months at baseline and 794 children (446 intervention and 348 at controlled group) aged 5 years at follow-up.	5 years	The oral health promotion was emphasized by the public paediatric health nurse and by the dental personnel at the ages of 6–8 months, and later at 18 months. At these OHP visits, the main topics were dental health; oral bacteria and transmission pathways; planned regular meals; avoiding sugar; choosing healthy non-cariogenic food, drink, and snacks; oral hygiene; adequate use of fluorides; the development of teeth; and sucking habits.	No intervention	The proportion of children with dental caries (dmft> 0) at 5 years old. Dental biofilm samples were also taken from maxillary incisors and using dental floss.	OHP can reduce the risk for caries in white-collar families than in blue-collar families, which **increased** the caries inequalities.<br><br>OHP was effective in white-collar families [numbers needed to treat (NNT) = 3, 95% CI 2-11]. Factors significantly associated with caries at 5 years were MS colonization at 18 months, occupation of caretaker, but also gender when incipient carious lesions were included in the index.

			Caretakers received a toothbrush for the child. The intervention, targeted to *Mutans Streptococci* positive subjects in the intervention group, was based on repeated **health education** to the caretakers and xylitol lozenges for the child.			
Ellwood (2004) United Kingdom [17]	7422 children aged 1 year old at baseline and 3467 children aged 5-6 years old at follow-up	5 years	**Fluoride toothpaste** containing either 440 or 1450 ppm F and dental health literature posted at 3-month intervals and toothbrush provided annually from the age of 1–5.5years.	Comparison group received no intervention	Mean dmft and proportion of participants with dmft > 0, dmft >4, and upper primary incisor caries. SES was assessed using Townsend index of material deprivation.	The program **did not reduce** area deprivation inequalities (**no effect**).<br><br>Participants in the programme using the high-fluoride toothpaste had significantly ($P < 0.002$) less caries than the comparison group with similar absolute reductions in mean dmft for the most- and least-deprived groups. Relative to the comparison group the association between deprivation and dental caries was changed so that in the most-deprived quartile those using the low-fluoride toothpaste tended to have less dental caries than the comparison group whereas in the least deprived they tended to have more ($P < 0.05$).
Matsuo (2020) USA	1872 children aged 10-19 years old (1057 in water fluoridation area and 815		**Water fluoridation** area	No water fluoridation area	Carious lesions according to the WHO as	Community water fluoridation (CWF) **reduced dental caries and its disparities**. The results of this study support the inclusion of CWF as a primary public health

	in non-water fluoridation area).				non-cavitated lesions and cavitated lesions. The DMFS for examiner was dichotomized as no caries surfaces (DMFS=0) vs some caries surfaces (DMFS>=1).	intervention to reduce dental caries and oral health disparities.
Faustino-Silva (2019) Brazil [12]	915 children at baseline and 414 children (186 were included in the CE group and 228 in the MI group) at follow-up	1.7 years (49 days-3.8 years)	**Motivational interviewing (MI)** In the MI group, the oral health teams (OHT) attended two 4 - hour-training sessions. In the MI group, the OHTs acted in accordance with the spirit and techniques of this approach, using empathic communication skills and using Simple and Complex Reflexive Listening to work with resistance and ambivalence, developing discrepancy, listening and encouraging change talk.	**Conventional health education** Both oral health teams (OHT) acted according to service protocol, what differentiated intervention and conventional education during the visits that took place in the children's first year of life was the way in which advice	The clinical examination of caries was recorded through ICDAS, which was converted into dmfs index for analysis.	Motivational interviewing had a greater preventive effect against caries in lower income children, which **reduced** the caries inequalities. The effect of MI was statistically significant in the lower income category (*P*=0.03); MI prevented 57% of carious lesions (IRR: 0.43, 95% CI 0.22 - 0.83) and reduced the occurrence of the disease on more than one surface per 100 followed surface - year in this same category in the equivalent income subgroup (IRD: −1.37, *P*=0.04).

				was delivered.		
Kim (2017)<br>South Korea<br>11	1313 children (627 in non-CWF area and 686 in CWF area) aged 6, 8 and 11 years old		**Water fluoridation** area	No water fluoridation area	DMFT index was assessed with WHO criteria for oral health surveys	The results suggest that CWF programs are effective in the prevention of caries in permanent teeth among children and can **reduce** oral health inequalities indicated by family income, affluence score and caregiver education level.
McLaren (2016)<br>Canada<br>18	557 children in 2009/2010 and 3230 children in 2013/2014	4 years	After cessation of community of **water fluoridation**	Before cessation of community of water fluoridation	The number of deft and DMFT. Those with two or more teeth with untreated decay as a dichotomous variable (yes vs. no).	The results showed an increase deprivation inequities (small area index) in dental caries following cessation of CWF. This implies that applying water fluoridation is useful in **reducing** caries inequities.
Sagheri (2007)<br>Ireland<br>13	699 children aged 12 years old (322 in Freiburg and 377 in Dublin).		**Water fluoridation** area	No water fluoridation area	Dental caries was recorded at the level of cavitation into dentine using WHO criteria.	The evidence from this study confirmed that water fluoridation has **reduced** the gap in dental caries experience between medium and lower social classes in Dublin compared with the greater difference in caries experience between the equivalent social classes in Freiburg.
